# Valvulo-Arterial Impedance and Dimensionless Index for Risk Stratifying Patients With Severe Aortic Stenosis

**DOI:** 10.3389/fcvm.2021.742297

**Published:** 2021-12-02

**Authors:** Yogamaya Mantha, Shutaro Futami, Shohei Moriyama, Michinari Hieda

**Affiliations:** ^1^Division of Cardiovascular Medicine, University of Texas Health Sciences, San Antonio, TX, United States; ^2^Department of Medicine and Biosystemic Science, Hematology, Oncology and Cardiovascular Medicine, School of Medicine, Kyushu University, Fukuoka, Japan

**Keywords:** aortic stenosis, valvulo-arterial impedance, dimensionless index, paradoxical low-flow low-gradient severe AS, heart failure, valvular heart disease, global load, echocardiography

## Abstract

The hemodynamic effects of aortic stenosis (AS) consist of increased left ventricular (LV) afterload, reduced myocardial compliance, and increased myocardial workload. The LV in AS patients faces a double load: valvular and arterial loads. As such, the presence of symptoms and occurrence of adverse events in AS should better correlate with calculating the global burden faced by the LV in addition to the transvalvular gradient and aortic valve area (AVA). The valvulo-arterial impedance (Zva) is a useful parameter providing an estimate of the global LV hemodynamic load that results from the summation of the valvular and vascular loads. In addition to calculating the global LV afterload, it is paramount to estimate the stenosis severity accurately. In clinical practice, the management of low-flow low-gradient (LF-LG) severe AS with preserved LV ejection fraction requires careful confirmation of stenosis severity. In addition to the Zva, the dimensionless index (DI) is a very useful parameter to express the size of the effective valvular area as a proportion of the cross-section area of the left ventricular outlet tract velocity-time integral (LVOT-VTI) to that of the aortic valve jet (dimensionless velocity ratio). The DI is calculated by a ratio of the sub-valvular velocity obtained by pulsed-wave Doppler (LVOT-VTI) divided by the maximum velocity obtained by continuous-wave Doppler across the aortic valve (AV-VTI). In contrast to AVA measurement, the DI does not require the calculation of LVOT cross-sectional area, a major cause of erroneous assessment and underestimation of AVA. Hence, among patients with LG severe AS and preserved LV ejection fraction, calculation of DI in routine echocardiographic practice may be useful to identify a subgroup of patients at higher risk of mortality who may derive benefit from aortic valve replacement. This article aims to elucidate the Zva and DI in different clinical situations, correlate with the standard indexes of AS severity, LV geometry, and function, and thus prove to improve risk stratification and clinical decision making in patients with severe AS.

## Introduction

Aortic stenosis (AS) is one of the most common and critical valve diseases in the world. In North America and Europe, aortic valve disease is primarily due to calcification or a congenital bicuspid valve. AS progresses with aging as calcium, thickening, inflammation, or scarring damages the valve, which restricts blood flow. The normal aortic valve area is between 3.0 and 4.0 cm^2^. The pressure gradient across the aortic valve and aortic transvalvular flow is directly correlated to the aortic valve area. In general, patients with AS will have symptoms of heart failure when the aortic valve area is <1.0 cm^2^ and/or the mean pressure gradient is over 40 mmHg (Table 1). The pressure gradient utilizing the modified Bernoulli equation indicates a robust correlation with direct pressure measurement.


$$\begin{array}{l} \text{Δ}P\;=4\;\times {\left(V_{AV}\right)}^{2} \end{array}$$

**Table 1:** Hemodynamic parameters for assessment of aortic stenosis and their cutoff values for severe aortic stenosis.

**Hemodynamic parameters**	**Method**	**Severe AS cutoff**
Aortic stenosis jet velocity	Direct measure	>4.0 m/s
Mean pressure gradient	Direct measure (Cath) Bernoulli equation (Echo)	>40 mmHg
Aortic valve area (AVA)	Gorlin equation (Cath) Continuity equation (Echo)	<1.0 cm^2^
Indexed AVA	EOA normalized by BSA	<0.6 cm^2^/m^2^
Dimensionless index (DI)	Ratio of LVOT-VTI and AV-VTI	<0.25
Valvulo-arterial impedance (Zva)	Global systolic LV afterload, including arterial pressure	4.5–5.0 mmHg/ml/m^2^
Energy loss index	Indexed EOA accounting for ascending aorta size	<0.5–0.6 cm^2^/m^2^
Aortic valve resistance	Resistance of AV to flow	>280 dynes s cm^−5^
Calcium score	Measured from CT data	>1,651 AU

**Figure 1:**
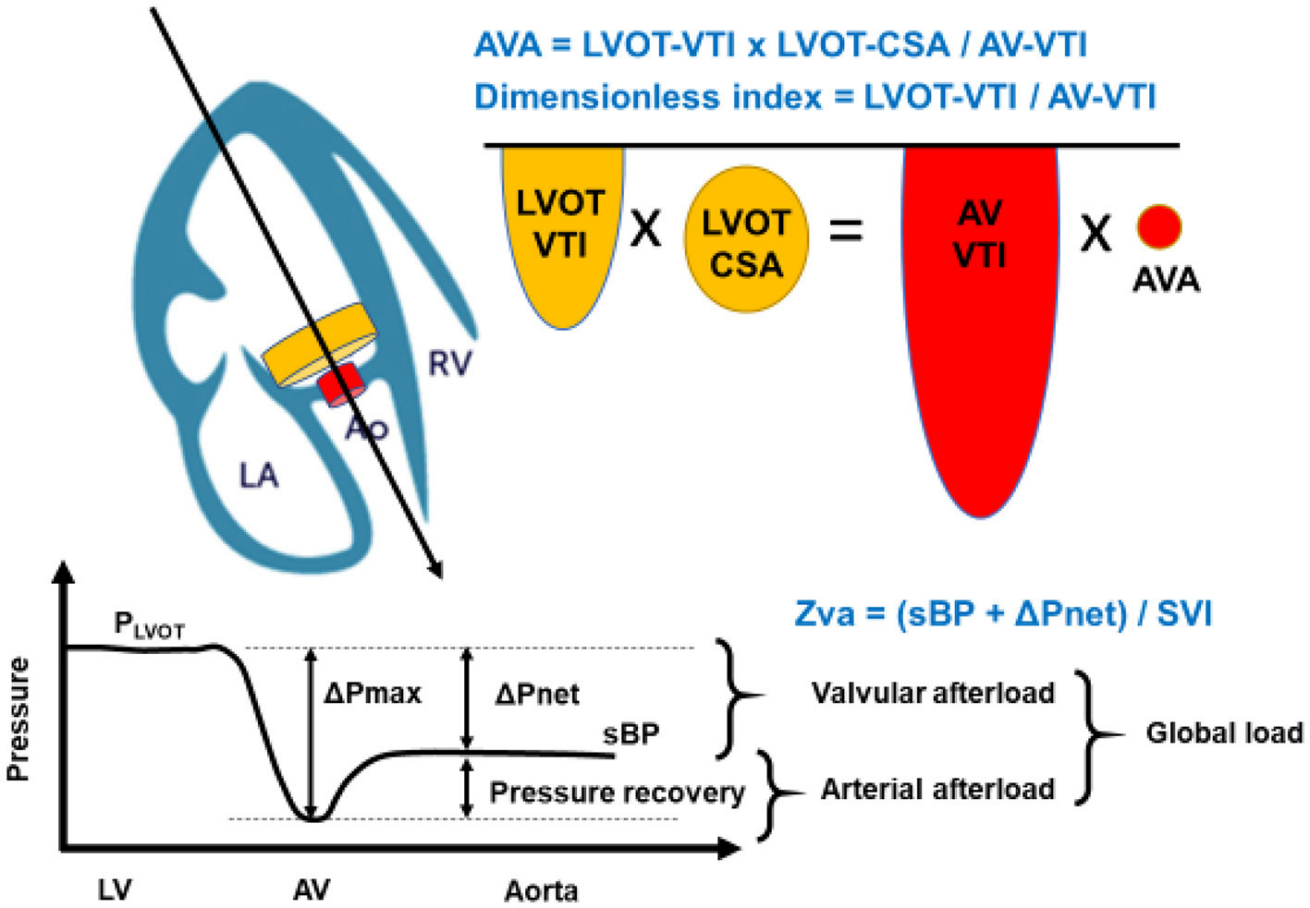
Concept of Aortic Valve Area (AVA) calculation, dimensionless index, and Zva. The calculation of AVA is a standard and must be incorporated into a comprehensive evaluation of aortic stenosis severity. The angle of color Doppler should be accurately aligned. As the LVOT radius is squared to obtain LVOT-CSA in AVA calculation, which may allow inaccuracies and can also contribute substantially to error. The dimensionless index is obtained by LVOT-VTI divided by AV-VTI. The global hemodynamic load imposed on the LV results from the summation of the valvular afterload and the arterial afterload. This global load can be estimated by calculating the valvulo-arterial impedance (Zva). Zva can be calculated with the Doppler mean pressure gradient in place of the ΔPnet: mean pressure gradient. LA, left atrium; Ao, aortic valve; RV, right ventricle; AVA, aortic valve area; LVOT-VTI, left ventricular outlet tract- velocity time integral; LVOT-CSA, cross sectional area; AV-VTI, aortic stenosis- velocity time integral; Zva, valvulo-arterial impedance; sBP, systolic blood pressure; SVI, stroke volume index.

*AS, aortic stenosis; Cath, Catheter; Echo, echocardiography; EOA, effective orifice area; AV, aortic valve; Cath, catheter; Echo, echocardiography; BSA, body surface area; LVOT, left ventricular outlet tract; VTI, velocity-time integral; CT, computer tomography; AU, Agatston unit*.

where ΔP (mmHg) is the maximum pressure gradient between the left ventricle (LV) and the aorta, V_AV_ (m/s) is the maximum stenotic jet velocity. Then, the pressure gradient is applied to estimate the aortic valve area by the continuity principle.


$$\begin{array}{lll} Flow\;volume & = & LVOT\text{-}VTI\;\times \;LVOT\text{-}CSA\\ \; & = & \;AV\text{-}VTI\;\times \;AVA \end{array}$$

where LVOT-VTI is velocity-time integral in the left ventricular outflow tract, LVOT-CSA is the cross-sectional area of the left ventricular outflow tract, AV-VTI is velocity-time integral across the aortic valve, and AVA is the area of the stenotic aortic valve (Figure 1). Although the severity of AS can be assessed by Doppler echocardiography in almost all patients, it may be underestimated if the range of the interest in Doppler is not well-aligned with the AS jet. Moreover, these conventional methods are limited in low-flow states. As such, the severity of the stenosis may be underestimated in patients with lower left ventricular ejection fraction (LVEF) and cardiac output.

In addition, AS is not just a disease of the valve itself. Indeed, AS is a complex systemic disease with abnormalities of the systemic arterial system such as reduced arterial compliance, LV systolic dysfunction, and reduced transvalvular flow rate, which pose important challenges with regards to diagnostic evaluation and clinical decision making in AS patients (1, 2). Hence, the assessment of AS severity, as well as its therapeutic management, should be conducted with the use of a comprehensive evaluation of the aortic valve, myocardial compliance, and the global LV hemodynamic afterload. The pathophysiology of adverse outcomes in AS is essentially due to an imbalance between the increase in LV hemodynamic afterload due to the valvular obstruction and/or concomitant arterial hypertension, and the capacity of the LV to overcome the “double load.” To reiterate, a double load imposed on the left ventricle by AS, in addition to an increased arterial afterload, the LV hypertrophic process is accelerated due to an aggregated increase in thickness of the LV wall (3). According to the current guidelines, valvular replacement is indicated for severe symptomatic AS and for some groups in asymptomatic individuals with severe AS and preserved LVEF (4–6). In particular, the transvalvular velocity, pressure gradient, and aortic valve area are highlighted in the severity of AS. In this review, we summarize the hemodynamics of severe AS, global LV load, valvulo-arterial impedance (Zva), and accurate assessment of severity with dimensionless index (DI) in different clinical situations, which might aid in improving the prognosis of AS (Figure 1, Table 2).

**Table 2:** Comparison of AS parameters.

	**AVA by continuous equation**	**Valvulo-arterial impedance (Zva)**	**Dimensionless index**
Advantage	✓ Easy to understand ✓ Easy to follow up	✓ Enable to consider the actual global hemodynamic afterload (valvular + arterial afterload) for evaluation	✓ Easy to calculate ✓ Easy to follow up ✓ Unnecessary to measure the LVOT-CSA ✓ Enable to distinguish the pressure gradient (AS vs. LVOT-stenosis)
Disadvantages	✓ Need to measure LVOT-CSA, which may lead to be inaccuracy (the shape of LVOT is not perfect circle) ✓ Both LVOT-VTI and AV-VTI are depended on the Doppler angle ✓ Sigmoid septum and LVOT stenosis may influence the VTIs ✓ Dependent on technical skill of sonographer	✓ Both LVOT-VTI and AV-VTI are depended on the Doppler angle ✓ Need to measure systolic blood pressure, carefully ✓ Dependent on technical skill of sonographer	✓ Both LVOT-VTI and AV-VTI are depended on the Doppler angle ✓ Dependent on technical skill of sonographer

*AS, aortic stenosis; AVA, aortic valve area; LVOT-CSA, left ventricular outlet tract- cross sectional area; VTI, velocity time integral; Zva, valvulo-arterial impedance*.

## Assessment of Aortic Stenosis and Hemodynamics

According to the current American Heart Association/American College of Cardiology and 2021 European Society of Cardiology/European Association of Cardio-thoracic Surgery (ESC/EACTS) guidelines, echocardiographic evaluation of severe AS is defined as: peak transvalvular velocity (Vpeak) ≥ 4.0 m/s, mean pressure gradient (mPG) ≥ 40 mm Hg, aortic valve area (AVA) ≤ 1.0 cm^2^ and/or AVA indexed for body surface area (BSA) ≤ 0.6 cm^2^/m^2^ (4, 6, 7). Indexing AVA for BSA is important in patients whom the valve area may be small but not severely stenotic when adjusted for body size. Discrepancies are frequently encountered between mPG or Vpeak and AVA (8). Moreover, the calculation of the size of the functional aortic orifice by the continuity equation relies on the accurate measurement of the LVOT-CSA (9), frequently underestimated by echocardiography (10) (Table 3). Studies have shown that the LVOT is often elliptical and not circular and thus measuring the LVOT diameter in patients with severe valve calcification is challenging (12, 13). In addition, accurate recording of peak aortic jet velocity requires parallel alignment between the continuous Doppler ultrasound beam and the aortic flow. Non-parallel alignment leads to underestimation of AS severity and thus is operator dependent (2). In clinical practice, these discordant findings may lead to inaccurate assessment of the severity of the AS that could delay therapeutic management and thus produce negative patient outcomes.

**Table 3:** Stages of aortic stenosis.

**Stage**	**Description and symptoms**	**Valve anatomy**		**Hemodynamics**		**LVEF**
		**Calc**	**Mobility**	**Key criteria**	**Additional measures**	
A	At risk (asymptomatic)	+	Normal	Aortic Vmax < 2 m/s	–	Normal
B	Progressive (asymptomatic)	++	↓ to ↓↓	Mild AS: Aortic Vmax 2.0–2.9 m/s or mean ΔP < 20 mmHg	–	Normal
				Moderate AS: Aortic Vmax 3.0–3.9 m/s or mean ΔP 20–39 mmHg		
C1	Asymptomatic severe AS with normal LVEF	+++	↓*↓↓*	Aortic Vmax ≥ 4 m/s or mean ΔP 40 mmHg (severe)	AVA typically ≤ 1 cm^2^ (or AVAi ≤ 0.6 cm^2^/m^2^)	Normal
				Aortic Vmax ≥ 5 m/s or mean ΔP ≥ 60 mmHg (very severe)		
C2	Asymptomatic severe AS with low LVEF	+++	↓*↓↓*	Aortic Vmax ≥ 4 m/s or mean ΔP ≥ 40 mmHg (severe)	AVA typically ≤ 1 cm2 (or AVAi ≤ 0.6 cm^2^/m^2^)	<50%
D1	Symptomatic severe high-gradient AS	++++	↓*↓↓↓*	Aortic Vmax ≥ 4 m/s or mean ΔP ≥ 40 mmHg	AVA typically ≤ 1 cm2 (or AVAi ≤ 0.6 cm^2^/m^2^) but may be larger with mixed AS/AR	Normal or ↓
D2	Symptomatic severe low-gradient AS with low LVEF	++++	↓*↓↓↓*	Resting AVA ≤ 1 cm^2^ with aortic Vmax < 4 m/s or mean Δ*P* < 40 mmHg	Dobutamine stress shows AVA ≤ 1 cm^2^ with Vmax ≥4 m/s at any flow rate	<50%
D3	Symptomatic severe low-gradient AS with normal LVEF	++++	↓*↓↓↓*	AVA ≤ 1 cm^2^ with aortic Vmax < 4 m/s or mean Δ*P* < 40 mmHg Measured when the patient is normotensive (systolic BP < 140 mmHg)	Indexed AVA ≤ 0.6 cm^2^/m^2^ and stroke volume index < 35 mL/m^2^	Normal

*Calc, calcification; LVEF, left ventricular ejection fraction; +, present, with severity indicated by number of symbols; Vmax, maximum transvalvular aortic velocity; ↓, decreased, with degree indicated by number of arrows; AS, aortic stenosis; ΔP, pressure gradient; AVA, aortic valve area; AVAi, valve area indexed for body surface area; AR, aortic regurgitation; BP, blood pressure. Otto CM, Nishimura RA. New ACC/AHA valve guidelines: aligning definitions of aortic stenosis severity with treatment recommendations. (11); 100:902*.

## Valvulo-Arterial Impedance (Zva) as Global Left Ventricular Hemodynamic Load

Hypertension is found in one-third of patients presenting with symptomatic severe AS. The resultant LV remodeling and hypertrophy are adaptive responses to chronic LV systolic pressure overload and are commonly encountered in patients with hypertension and AS. There are several mechanisms that can explain the important reduction of mean gradient in the presence of hypertension at any AS severity; (1) hypertension causes an increase in systemic vascular resistance, and (2) the decrease in stroke volume induced by increased afterload leads to a reduction in transvalvular gradient and velocity. Thus, the increased “double load,” which includes the arterial and LV afterload, interferes with Doppler echocardiographic evaluation of the severity of AS (Figure 1).

In order to negate the falsely low transvalvular gradient and velocity, the valvulo-arterial impedance index (Zva) can be used (14). Zva is a measure of the global LV afterload. In patients with AS, the hemodynamic index is defined as the ratio of the LV systolic pressure over stroke volume indexed (SVI) to BSA, that is,


$$\begin{array}{l} Zva=\frac{\left(sBP+\text{Δ}Pnet\right)}{SVI} \end{array}$$

where sBP is the systolic blood pressure, and ΔPnet is the mean transvalvular pressure gradient after pressure recovery (14). Thus, this Zva can estimate global LV load and represents the cost in mmHg for each systemic milliliter of blood indexed for BSA pumped by the LV during systole (Figure 1). The index can be calculated via non-invasive means such as the Doppler echocardiogram or invasive means with cardiac catheterization. According to a previous study, a value of Zva ≥ 5.0 mmHg/mL·m^2^ might represent LV afterload mismatch, and LV systolic dysfunction and Zva ≥ 5.5 mmHg/mL·m^2^ was associated with a 2.5-fold increase in the risk of overall mortality (15). Moreover, the Zva is particularly useful in patients who do not meet the criteria of severe AS such as with low-flow, low-gradient (LF-LG) severe AS and preserved LVEF. Zva was also the main determinant of LV dysfunction in asymptomatic paradoxical LF-LG severe AS in the SEAS study (16). Similar findings were demonstrated by Hachicha et al. with increased Zva (>3.5 mmHg/mL·m^2^) as a predictor of poor outcome in asymptomatic severe AS patients due to associated LV systolic and diastolic dysfunction (17). These data suggest that Zva may guide risk stratification and therapeutic management in AS patients who do not fit the criteria for typical severe AS.

## Dimensionless Index

The dimensionless index (DI) represents the ratio of the LVOT VTI in relation to that of the aortic valve jet by eliminating the highly erroneous measurement of LVOT cross-sectional area from the continuity equation (18).


$$\begin{array}{l} Dimensionless\;Index\;\left(DI\right)=\frac{LVOT\text{-}VTI}{AV\text{-}VTI} \end{array}$$

The DI is calculated by a ratio of the sub-valvular velocity obtained by pulsed-wave Doppler (LVOT-VTI) divided by the maximum velocity obtained by continuous-wave Doppler across the aortic valve (AV-VTI) (19) (Figure 1). It expresses the size of the valvular effective area as a proportion of the CSA of the LVOT. Alternatively, the ratio of the peak velocity of LVOT to peak AV jet can be used. In the absence of valve stenosis, the velocity ratio approaches 1, with smaller numbers indicating severe stenosis. For instance, severe AS is present when the velocity ratio is 0.25 or less, representing 25% of the valve. Otto et al. reported that DI showed better sensitivity than Doppler pressure gradient to identify severe AS (97 vs. 81%) (20). DI is normalized for body size as it reflects the ratio of the actual valve area compared to the expected valve area.

**Figure 2:**
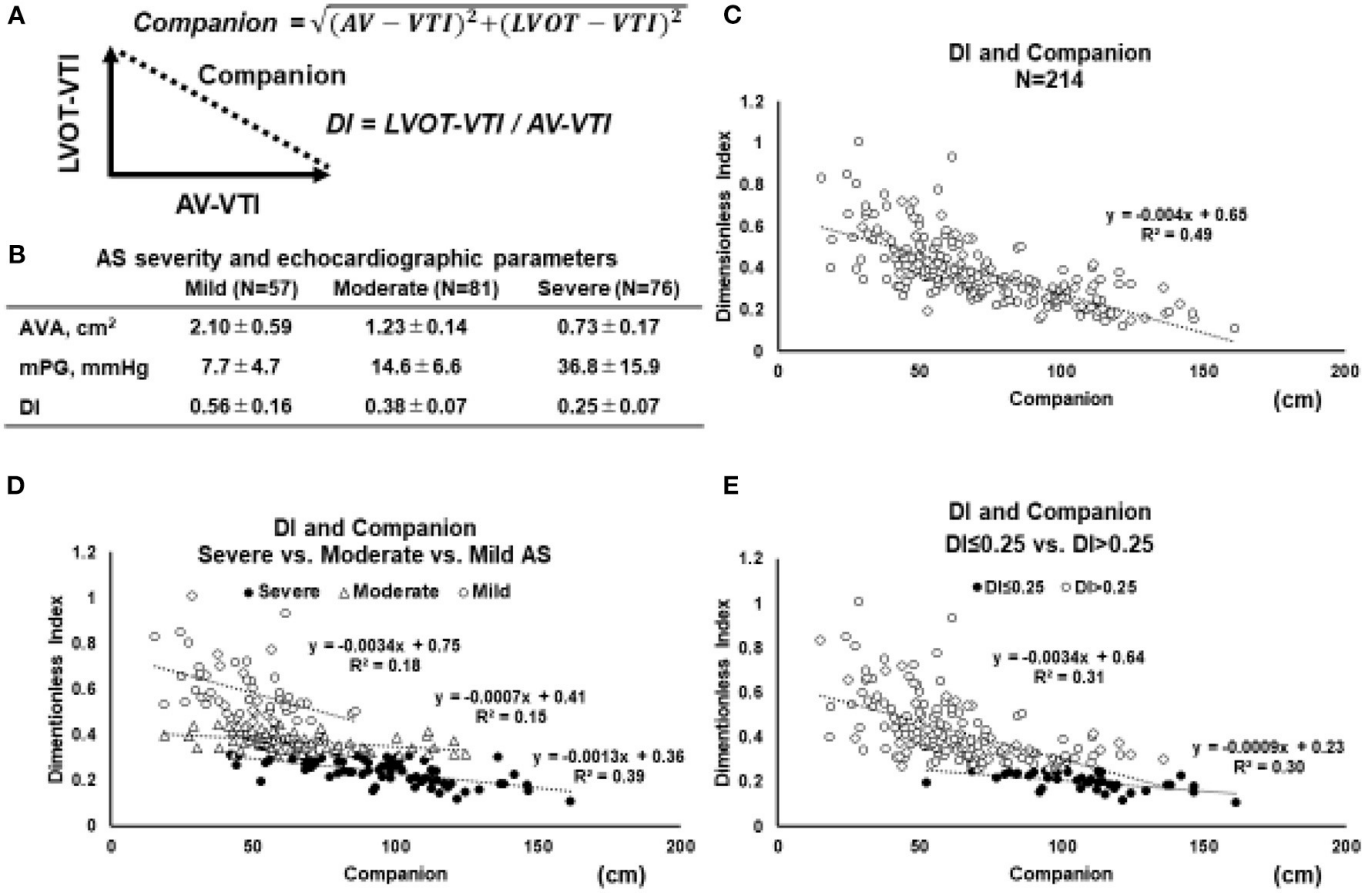
Relation between DI and its companion. **(A)** Geometric relationship between the two parameters and the companion. Based on Pythagorean theorem, the companion (diagonal length of the triangle) is obtained by the following equation: Companion = √(*AV* − *VTI*)^2^ + (*LVOT* − *VTI*)^2^. **(B)** In order to elucidate the behavior of the relation between DI and companion, the echocardiographic parameters in 214 patients with AS are retrospectively reviewed. **(C)** The relations between the DI and companion in whole AS patients (*N* = 214). **(D)** The relation between the DI and companion in separated by the severity of AS (mild vs. moderate vs. severe). **(E)** The relation between the DI and companion in AS patients with DI ≤ 0.25 vs. DI > 0.25 **(E)**. Abbreviations are as same as in Figure 1.

In cardiovascular research, ratio-based indices are often used, such as LVEF (21), arterial pressure augmentation index (22), coronary fractional flow reserve (23), and pulse pressure (24). When the ratio is used alone, it may provide incomplete information about the content of the two components of a fraction-based index. Indeed, just focusing on the single number of the ratio results in a loss of information. However, the lost information can be regained without additional measurements by applying the Pythagorean mean (25). A polar coordinate description can reveal the companion of the traditional metric. The clinical relevance of both the ratio and the associated companion can often be clarified by demonstrating a statistically significant association with an established physical measure with sound interpretation. In the case of the DI, constitutive metrics are AV-VTI and LVOT-VTI. Companion (the diagonal length of the triangle) is calculated using the Pythagorean theorem (Figure 2A).


$$\begin{array}{l} Companion=\sqrt{{\left(AV\text{-}VTI\right)}^{2}+{\left(LVOT\text{-}VTI\right)}^{2}} \end{array}$$

To elucidate the behavior of the relation between DI and its companion, echocardiographic parameters in 214 patients with AS are retrospectively reviewed (Figure 2B). The relation between the DI and companion in whole AS patients (*N* = 214) (Figure 2C), separated by the severity of AS (mild vs. moderate vs. severe) (Figure 2D), and in patients with DI ≤ 0.25 vs. DI > 0.25 (Figure 2E). According to the 2021 ESC/ EACTS guidelines, DI value of < 0.25 suggests severe AS, especially when other clinical and echocardiographic parameters are equivocal (6). When stratified by AS severity, it was found that each group could be separated relatively clearly (Figure 2D). These figures indicated that the smaller the DI, the more the slope asymptotes to zero, regardless of the companion. Especially in the AS patients with DI < 0.25, the slope became almost parallel to the x-axis, and the triangle formed by AV-VTI and LVOT-VTI changed with a constant similarity ratio. Taken together, the DI can separate AS severity with clarity and enables clinicians to assess the severity of AS without worrying about the magnitude of AV-VTI and LVOT-VTI if it is <0.25.

## Asymptomatic Severe as

Asymptomatic severe AS is classified into the two groups: (1) asymptomatic severe AS with normal LVEF (Stage C1) and (2) asymptomatic severe AS with low LVEF (Stage C2) (5) (Table 3). Surgical intervention is indicated for patients with asymptomatic severe AS (Stage C2). In patients with asymptomatic severe AS, the determinant factors resulting in poor prognosis are higher peak velocity (≥5 m/s) (26), rapid progression of stenosis (incremental peak velocity > 0.3 m/s/year) (27, 28), elevated BNP (29), and pulmonary hypertension (tricuspid valve regurgitation velocity ≥3.5 m/s or systolic pulmonary arterial pressure ≥60 mmHg) (30, 31). Current guidelines recommend that patients with asymptomatic severe AS including these parameters and lower risk of surgery should have AVR (5, 32). One of the additions in the 2021 ESC/ EACTS guidelines include surgery, as a class I recommendation, for asymptomatic patients resting LVEF ≤ 50% without another differential diagnosis. In addition, early intervention in asymptomatic patients (Class IIa) with a LVEF < 55% and a normal exercise test if the stenosis is severe; i.e. mean gradient > 60 mmHg or Vmax >5 m/s, severe calcification on cardiac computed tomography and Vmax progression > 0.3 m/s/year, or elevated BNP that is three times the normal range (6). In addition, moderate to severe calcification of the aortic valve (28), reduced global longitudinal strain (GLS) (33), and impaired left atrial function (left atrial dilatation and reduced peak late diastolic annular velocity) have also been reported to be risk factors of AS progression (29, 34). Therefore, early and appropriate surgical aortic valve replacement (AVR) is recommended for high-risk severe AS patients in the stage C1, rather than clinical observation (26, 35, 36).

Several studies have demonstrated favorable impacts of DI and Zva on the clinical management of patients with asymptomatic severe AS (18, 37, 38). In the cohort of asymptomatic and minimally symptomatic severe AS, lower DI was significantly associated with poor mortality (18, 38). Rusinaru et al. reported that the risk of all-cause mortality and AVR increased by about 25% for every 0.05 decrease in the DI (18). Interestingly, patients with very low DI (< 0.2) had particularly increased adverse events, compared to that of severe AS and higher DI (18, 39). Oh et al. also reported that low DI (DI ≤ 0.25) was significantly related to the higher severity of AS (AVA ≤ 0.75 cm^2^) measured by cardiac catheterization (40). In addition, the combination of DI, AVA, and AV peak velocity was proven to be more sensitive indicators of cardiac adverse events, compared to AVA alone or a combination of AVA and AV peak velocity (18). Thus, the combination of DI and other hemodynamic parameters can be a promising clinical predictor for the timing of AVR.

Increased Zva is a useful predictor of LV hemodynamic afterload (37, 41). These myocardial changes instigate cardiac diastolic and systolic dysfunction, symptomatic heart failure, and cardiovascular death (37, 41–44). In addition, it is becoming evident that a higher global LV load which Zva quantifies is associated with the progression of LV dysfunction. Cramariuc et al. demonstrated that abnormal stress corrected mid-wall shortening and LV longitudinal strain was seen in 10% of patients in the lowest vs. 63% of patients in the highest tertile of Zva (*P* < 0.001) in 1,591 patients with asymptomatic severe AS and normal LVEF in the Simvastatin Ezetimibe in Aortic Stenosis trial (16). Currently, Zva > 5.0 mmHg/mL·m^2^ is considered to be an indicator of severe AS (45). A previous study reported that Zva value of > 3.5 mmHg/mL·m^2^ was able to predict AS progression or mortality in the patients with asymptomatic AS (41). Taken together, Zva may be used to improve risk stratification and clinical decision-making in patients with asymptomatic AS. To determine the timing of surgical intervention, the appropriate value of Zva should be further investigated.

## Symptomatic Low Gradient Moderate as

Moderate AS is defined hemodynamically as an AV velocity of 3.0–3.9 m/s or mean pressure gradient (PG) of 20–39 mmHg, with AVA ≥ 1.0 cm^2^ or normal AV flow (SVI ≥ 35 L/m^2^) (5, 43). When a patient is classified as moderate AS with lower SVI (<35 mL/m^2^), additional examinations should be performed to assess whether the severity of AS is severe or moderate. In addition, if patients with moderate AS may have heart failure symptoms such as dyspnea on effort, chest pain, or fainting, the next two points need to be reconsidered: (1) Is the severity of AS really moderate, but not severe? and (2) Are there any other diseases related to the heart failure symptoms other than AS?

To assess the AVA, maximum AV velocity should be selected from the highest velocity signal in multiple echocardiographic windows (43). However, obtaining an appropriate view is sometimes difficult in patients with poor echocardiographic delineation due to obesity, emaciation, emphysema, artificial breasts, or post-breast surgery. In these patients, AS severity may tend to be underestimated. In addition, there may be a discordance between the estimated LVOT by echocardiogram and its actual area due to the elliptical geometry of the LVOT (46). In contrast, DI does not require measurement of LVOT size and is less variable than the estimated AVA. Moreover, because DI is just a ratio, DI has the potential to assess the true severity of AS in patients with poor imaging in the echocardiogram. When AS severity is classified as moderate even after using the DI method, other reasons for the symptoms should be considered.

Evaluation of LV outflow tract stenosis (LVOTS) is also important. Abnormal LV septal thickening is observed about 10% in patients with AS (46). For patients with LVOTS, medications such as beta-blocker and cibenzoline are useful (47). In the condition of moderate AS with LVOTS, the DI can be useful for assessing AS severity (43). Zva can represent the effective global afterload against the LV (15, 17) (Figure 1). Indeed, the Zva is an independent predictor of syncope in patients with AS, which suggests that Zva may be able to detect hemodynamic changes in patients with AS (48). Thus, in identifying the causes of symptoms in patients with moderate AS, DI can play an important role in assessing the true severity of AS. In addition, Zva also can provide an accurate evaluation of global LV afterload in patients with moderate AS and LVOTS.

In patients with symptomatic moderate AS, coronary artery disease (CAD) should be carefully assessed. In patients with AS, CAD events occur at a rate of up to 20% per 5 years (49). Management of CAD risk factors, such as hyperlipidemia, hypertension, or diabetic mellitus, does not necessarily impede the progression of AS, but can prevent cardiac ischemic events (50, 51). In current guidelines, surgical AVR for moderate AS should be considered when another cardiac surgery is required (recommendation: class 2b) (52). When coronary artery bypass graft (CABG) is suitable, surgicalAVR with CABG is an indication.

## Severe as With Preserved Ejection Fraction

In patients with severe AS and preserved ejection fraction, deciding between clinical observation vs. early intervention to AS remains disputable (28, 52). The clinical challenges are (1) to predict precise prognosis and (2) to determine the appropriate timing of intervention in asymptomatic patients with severe AS. Current guidelines recommend that AVR is appropriate in asymptomatic patients with very severe AS; however, the definition of severe AS based on peak aortic jet velocity (Vmax) remains unclear with a 5.0 m/s cutoff in ACC/AHA guidelines (7) and 5.5 m/s in European guidelines (4). Approximately 20% of patients with severe AS and preserved LVEF have Vmax in this range between 5.0 and 5.5 m/s (53). Therefore, additional clinical indicators to make the clinical decision must be evaluated.

Regarding the usefulness of DI for evaluating the severity of AS in patients with preserved EF, Otto et al. reported that the DI was more sensitive than aortic valve pressure gradient (AV-PG) in identifying severe AS (sensitivity 97 vs 81%) (20). Moreover, Oh et al. also demonstrated that DI < 0.25 was also useful in identifying severe AS (40). Rusinaru et al. evaluated the relation between DI value and 5-year survival free of the events including cardiovascular death or need for AVR in AS patients without symptoms (18). This study reported that the DI was a reliable marker for assessing the severity of AS and that DI < 0.25 was a useful parameter in predicting prognosis. Thus, the DI can assess the severity and predict the prognosis of AS in severe AS patients with preserved ejection fraction.

AS is affected by the global hemodynamic loads imposed on the LV and arterial afterload (14, 54). Especially in patients with degenerative AS, the coexistence of arteriosclerosis and hypertension is common, and these coexisting diseases increase systemic vascular resistance. Therefore, a comprehensive evaluation of the “actual” global hemodynamic load by Zva is even more useful than conventional AS parameters. Hachica et al. reported that Zva predicted adverse outcomes in asymptomatic AS patients with preserved LVEF, even after adjustment for standard indexes of LV geometry and function. Indeed, Zva > 3.5 mmHg/mL·m^2^ can detect a poor prognosis in these patients (45). Zito et al. also investigated the role of the Zva in patients of asymptomatic severe AS with preserved LVEF and demonstrated that Zva was independently associated with death, AVR, and heart failure symptoms including dyspnea, angina, and syncope (36). They also reported that Zva ≥ 4.7 mmHg/ml·m^2^ was identified as the best cutoff value associated with the cardiovascular events (sensitivity 100% and specificity 91%) (36). Based on these results, the Zva is a useful parameter for performing risk stratification and clinical decisions in severe AS with preserved LVEF.

## Low Flow, Low Gradient Severe as

Low-flow (LF) severe AS (SVI ≤ 35 mL/m^2^) and low-gradient (LG) severe AS (transvalvular mean PG ≤ 40 mmHg) are partially overlapping categories in severe AS (Table 4). Severe AS with PG < 40 mmHg due to a decrease in cardiac output (SVI ≤ 35 mL/m^2^) is referred to as LF-LG severe AS (55). The type of LF-LG severe AS is separated into two types: (1) classical LF-LG severe AS and (2) paradoxical LF-LG severe AS. (1) The classical LF-LG severe AS is often observed in patients with the decreased LVEF (<40%). (2) The paradoxical LF-LG AS is due to both the LV narrowing cavity and LV diastolic dysfunction, despite preserved LVEF (LVEF ≥ 50%). The assessment of the actual type of LF-LG severe AS is important to decide therapeutic strategies.

**Table 4:** Types of severe aortic stenosis.

	**Normal-flow, high-gradient**	**Reduced LVEF, low-flow, low-gradient**	**Preserved LVEF (paradoxical), low-flow, low-gradient**
LVEF, %	>50	<40	>50
Aortic valve area, cm^2^	≤ 1.0	≤ 1.0	≤ 1.0
Aortic valve area index, cm^2^/m^2^	<0.6	<0.6	<0.6
Mean pressure gradient, mmHg	>40	<40	<40
Stroke volume index, ml/m^2^	>35	<35	<35
Dimensionless index	<0.25	<0.25	<0.25
Zva, mmHg/mL m^2^	>4.5	>4.5	>4.5
LV end-diastolic diameter, mm	45–55	>50	<47
Relative wall thickness	>0.43	0.35–0.55	>0.50
Global longitudinal strain, %	16–20	<14	<14
Myocardial fibrosis	+	+++	++

*LVEF, center ventricular ejection fraction; Zva, Valvulo-arterial impedance; LV, center ventricular. +, sometime; ++, likely; +++, often*.

There are two main causes of LV systolic dysfunction in classic LF-LG AS: (1) impacts from a progression of AS *per se* and (2) miscellaneous myocardial impairments such as myocardial ischemia or cardiomyopathy. Given LV systolic function is impaired by increased afterload due to severe AS *per se*, the peak aortic velocity and mean pressure gradient can be categorized in a moderate subset because the aortic velocity depends on the reduced flow rate. The effective orifice area (EOA) may be misinterpreted as a smaller area because the LV cannot provide sufficient stroke volume to generate the inertial force required to maximize the aortic valve opening. Therefore, patients with LF-LG AS need to be tested with increased SV to accurately assess EOA. Interestingly, it is well-known that the prognosis of classical LF-LG severe AS is worse than that of normal flow high gradient severe AS (56, 57). Therefore, it is important to distinguish “true-AS” or “pseudo-AS” in patients with LF-LG severe AS. As a useful evaluation method, dobutamine stress-echocardiography is recommended in guidelines (58). Dobutamine stress-echocardiography or invasive hemodynamic study with dobutamine can also evaluate the LV contractile reserve, determining whether it is true-AS or pseudo-AS and predicting patients' prognosis.

No studies have evaluated the impact of DI on clinical outcomes in patients with classical LF-LG severe AS. In assessing the severity of classical LF-LG severe AS, the AVA may be underestimated due to the reduced LV contractility and low flow rate (43). As being the LVOT measurement error, AVA calculated by the continuity equation might lead to measuring incorrectly. Thus, a multiparametric assessment is crucial in these patients. An ancillary study showed that an ejection dynamics parameter, the ratio of the acceleration time to ejection time >0.36, was associated with an increased risk of mortality in patients with LG severe AS (59). Multiple factors such as LV geometry, function, and systolic blood pressure the influence acceleration time to ejection time ratio. Interestingly, Bradley et al. looked at the accuracy of echocardiographic measures of AS severity in 77,067 patients where they found that a multiparameter assessment including using peak velocity, mean gradient, and DI provided the best sensitivity (92%) and specificity (99%) for diagnosis of severe AS compared with any single measure alone including AVA (60). Furthermore, when considering the relation between DI and the companion (Figure 2), DI may indicate the true severity of AS regardless of the size of companion, that is, flow size. A detailed evaluation of this relation is necessary in the future. The measurement of projected AVA under the dobutamine stress-echocardiography in classical LF-LG AS has been proposed in clinical guidelines (43, 61). However, it also includes the same problem caused by the measurement error of LVOT. In this respect, the DI, which excluded the effect of LVOT-CSA, might be useful as a value to support the change in AVA.

As mentioned, the impaired LV function in severe AS is affected by both the valvular and the arterial afterload. Therefore, Zva in classic LF-LG AS is theoretically high, especially in patients with lower SVI. Lewy et al. evaluated Zva in patients with severe symptomatic severe AS with LVEF ≤ 40% (62). The study demonstrated that higher Zva was associated with reduced LVEF. In this study, the Zva of patients with low LVEF-LG severe AS had a high value of 5.6 ± 1.7 mmHg/mL·m^2^. The assessment of contractile reserve in classical LF-LG severe AS is also important for the prognosis evaluation. This study also compared contractile reserve with Zva at rest by dobutamine stress-echocardiography and reported that patients with contractile reserve tended to have higher Zva (5.8 ± 1.8 vs 5.3 ± 1.3 mmHg/ml·m^2^; *p* = 0.07). There was also no statistical significance difference in Zva between resting and during dobutamine stress. Theoretically, pseudo-AS has a lower valvular afterload than true-AS. Therefore, it is ideal to evaluate the valvular and arterial afterload separately to distinguish between true-AS and pseudo-AS. However, it is difficult to separate these afterloads by Zva at rest. The Zva is flow-rate dependent (both mean PG [ΔPnet] and SVI, which are components of Zva) and can be changeable, especially in severe AS patients with low flow (63). In addition, the reduced arterial afterload due to the vasodilatory effect of low dose dobutamine may account for the differences in Zva between “true-AS” and “pseudo-AS,” Therefore, it might be useful to evaluate the change of Zva under dobutamine stress-echocardiography to distinguish between pseudo and true severe AS. However, this should be investigated further in future studies.

## Paradoxical Low Flow, Low Gradient Severe as

Paradoxical LF-LG severe AS has the preserved LVEF, but with the peak aortic velocity of <4.0 m/s and an average PG of <40 mmHg, and a valve area of fewer than 1 cm^2^ (34, 43) (Table 4). Patients with paradoxical LF-LG severe AS are more common in the elderly adults and characterized by markedly LV concentric hypertrophy and decreased stroke volume. Occasionally, technical errors may occur in the calculation of AVA by echocardiography, so careful assessment should be applied when diagnosing the severity of AS. It also remains challenging to distinguish between true- and pseudo-AS in patients with paradoxical LF-LG severe AS.

In paradoxical LF-LG severe AS, the LVOT often may be near obstructed due to LV hypertrophy and/or sigmoid septum. Both LV sigmoid septum and LVOT stenosis can increase apparent LVOT-VTI, leading to underestimation of DI. Hence, in the assessment of the severity of severe AS, it is necessary to consider that the morphology of LVOT may cause measurement errors related to the AVA and pressure gradient. DI, an AS severity index excluding the LVOT geometry, can be valuable for evaluating paradoxical LF-LG severe AS precisely as an alternative approach for reducing the measurement errors of the LVOT area. Indeed, Altes et al. reported that DI < 0.25 was a reliable parameter in the long-term mortality prediction of paradoxical LF-LG severe AS with or without symptoms (38). Furthermore, the patients with paradoxical LF-LG severe AS and DI < 0.25 were similar outcomes to the patients with high-gradient severe AS (38). As described in the above sections and Figure 2, DI may show the true severity regardless of the flow size. Therefore, further study in the LF-AS group is needed for the detailed evaluation.

Regarding Zva in paradoxical LF-LG AS, Hachicah et al. reported that Zva was higher than in patients with normal flow high gradient severe AS (17). In a multicenter prospective study, Adda et al. also reported that patients with paradoxical LF-LG severe AS had more severe AS (AVA 0.7 ± 0.12 vs. 0.86 ± 0.14 cm^2^) and higher Zva was seen in patients with normal-flow low-gradient AS (5.5 ± 1.1 vs. 4.0 ± 0.8 mmHg/mL·m^2^) (64). Those results suggested that the main mechanism of paradoxical LF-LG severe AS was elevated global afterload and that Zva may be useful in distinguishing the severity of paradoxical LF-LG AS. Regarding the effect of Zva on flow, unlike classical LF-LG AS, the SV index of preserved LF-LG AS is considered to be less affected by flow than that of classical LF-AS because the LVEF in paradoxical LF-LG AS is preserved, even if it is lower flow. Therefore, the influence of flow is considered smaller than that of classical LF-LG AS. However, as in the case of DI, the patient background of paradoxical LF-LG severe AS is sometimes accompanied by a sigmoid septum or LVOTS. This makes it difficult to assess the severity of AS because the ventricular outflow tract stenosis has an independent effect on left ventricular afterload. Therefore, a comprehensive evaluation of left ventricular geometry is required to assess the Zva in patients with paradoxical LF-LG AS.

## Conclusion

Clinical assessment of AS severity requires calculated AVA, mean and peak PG, and transvalvular flow. Although the AS severity can be assessed and diagnosed by echocardiography, it may be underestimated if echocardiography image quality is poor, especially if color Doppler images are not well-aligned with the AV jet. In addition, the accurate evaluation of AS severity is difficult to assess when cardiac output is lower and LVEF is reduced. The LVOT radius is squared to obtain LVOT-CSA in AVA calculation, allowing inaccuracies and contributing substantially to error. In order to resolve these limitations, the dimensionless index, which is a simple ratio of LVOT-VTI to AV-VTI with removing CSA from the simplified continuity equation, can be used. Among patients with LF-LG severe AS, the low flow can result in incomplete valve opening, leading to overestimate AS severity. Patients with LF-LG severe AS usually have elevated valvulo-arterial impedance (Zva), the assessment of AS severity in consideration with the Zva should be paramount. Incorporating dimensionless index and Zva with standard practices can improve risk stratification and clinical decision-making in patients with severe AS.

## Author Contributions

YM, SF, SM, and MH wrote sections of the manuscript. All authors contributed to manuscript revision, read, and approved the submitted version.

## Funding

MH was supported in part by Clinical Research Promotion Foundation. MH was also supported by JSPS KAKENHI Grant-in-Aid for Young Scientists Number 21K17603.

## Conflict of Interest

The authors declare that the research was conducted in the absence of any commercial or financial relationships that could be construed as a potential conflict of interest.

## Publisher's Note

All claims expressed in this article are solely those of the authors and do not necessarily represent those of their affiliated organizations, or those of the publisher, the editors and the reviewers. Any product that may be evaluated in this article, or claim that may be made by its manufacturer, is not guaranteed or endorsed by the publisher.
